# Pyranopyran-1,8-dione, an Active Compound from Vitices Fructus, Attenuates Cigarette-Smoke Induced Lung Inflammation in Mice

**DOI:** 10.3390/ijms18071602

**Published:** 2017-07-24

**Authors:** Gihyun Lee, Kyung-Hwa Jung, Eun Seok Ji, Hyunsu Bae

**Affiliations:** Department of Physiology, College of Korean Medicine, Kyung Hee University, 26 kyungheedae-ro, dongdaemoon-gu, Seoul 02447, Korea; glee@khu.ac.kr (G.L.); jhkh242@naver.com (K.-H.J.); ausomd@hanmail.net (E.S.J.)

**Keywords:** cigarette smoke, lung, inflammation, Viticis Fructus, phytochemical, pyranopyran-1, 8-dione

## Abstract

Previously, we isolated and identified pyranopyran-1,8-dione (PPY) from Viticis Fructus, as a bioactive compound possessing anti-inflammatory properties. The present study was aimed to evaluate the preventive benefit of PPY on cigarette–smoke (CS)-induced lung inflammation. C57BL/6 mice were exposed to CS for 2 weeks while PPY was administrated by oral injection 2 h before CS exposure. To validate the anti-inflammatory effects of PPY, the numbers of immune cells in the bronchoalveolar lavage fluid were counted. Proinflammatory cytokines (Tumor necrosis factor-α: TNF-α, IL-6) and keratinocyte chemokine (KC/CXCL1) were also measured. Histopathologic analysis and cellular profiles showed that inflammatory cell infiltrations were significantly decreased in peribronchial and perivascular area by PPY treatment. The alveolar destruction by CS was markedly ameliorated by PPY treatment. In addition, the TNF-α, IL-6, and KC levels were declined in the PPY groups. These observations suggest that PPY has a preventive potential for lung inflammatory diseases.

## 1. Introduction

**Figure 1:**
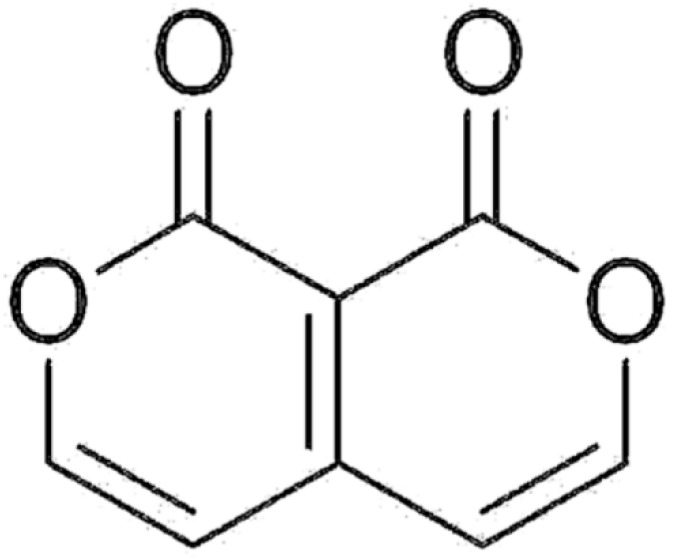
Structure of pyranopyran-1,8-dione.

During the last twenty years, new anti-inflammatory drugs are being discovered and developed as anti-cytokine agents with the massive expansion of the study on inflammatory response [1,2]. People, however, still long for cheaper and more effective alternative approaches to treat inflammation. Medicinal herbs have been used for treating inflammation related diseases, and they are often used to develop new medications [3,4,5,6]. It has been shown in a previous study that Fruit of *Vitex trifolia* subsp. *litoralis* Steenis, Viticis Fructus (VF), has anti-inflammatory properties, which occur via inhibition of the inflammatory cytokines and the mitogen-activated protein kinase (MAPK)-signaling pathway [5]. VF is widely used in China and Korea for the treatment of pain, cold, headache, migraine, sore eyes, myalgia, and gastrointestinal infections such as bacterial dysentery and diarrhea [7,8,9]. In one of our recent study, VF extract prevented the development of airway remodeling in asthma [10]. Therefore, we attempted to isolate the active compound from VF. After several rounds of activity-guided fractionations, pyranopyran-1,8-dione (PPY) was isolated (Figure 1) [11]. We previously reported that PPY inhibits inflammation in the ovalbumin-sensitized asthma mouse model [11]. Here, we used the CS-exposed mouse model for better understanding of the biomedical benefits of PPY, a natural chemical isolated from VF, on lung inflammation. In this study, we provide the evidence of PPY as a potent protective agent on lung inflammation.

## 2. Results

### 2.1. PPY Reduced Levels of Proinflammatory Cytokines and Chemokine KC in Bronchoalveolar Lavage (BAL) Fluid

**Figure 2:**
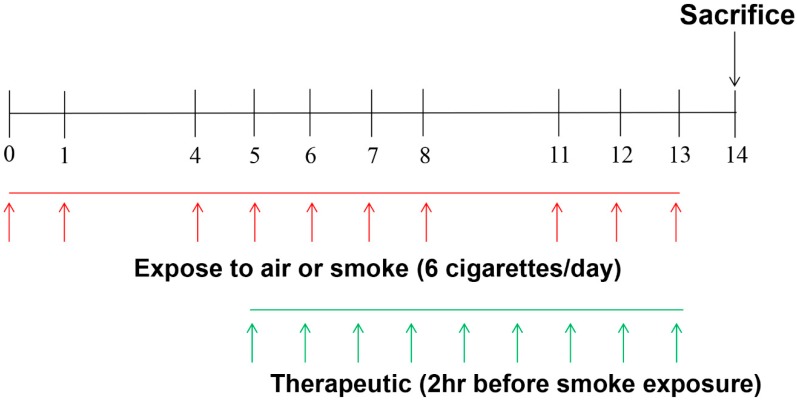
Schematic diagram of the experimental protocol Balb/c mice were exposed to cigarette smoke (CS, 6 cigarettes/day on day 0, 1, 4, 5, 6, 7, 8, 11, 12, and 13). For therapeutic study, vehicle, roflumilast (5 mg/kg), or PPY (1, 2 and 10 mg/kg) were administered 2 h before CS exposure from day 5 to 13. The mice were killed on day 14.

**Figure 3:**
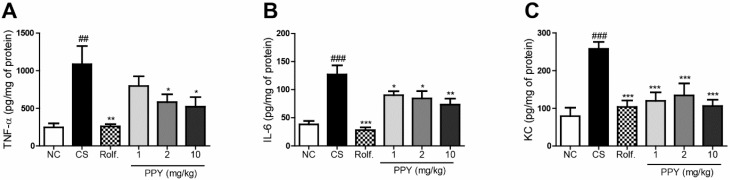
The effect of PPY on proinflammatory cytokine (TNF-α, IL-6) and KC levels in BAL fluid. Pro-inflammatory cytokine (TNF-α, IL-6) and CXCL-1 (KC) productions were measured using ELISA in the BAL fluid. (**A**) TNF-α; (**B**) IL-6 and (**C**) KC. Data are shown as mean ± S.E.M. Statistical analyses were conducted by one-way analysis of variance (ANOVA) followed by Newman–Keuls Multiple Comparison test (### *p* < 0.001, ## *p* < 0.01 vs. NC, *** *p* < 0.001, ** *p* < 0.01, * *p* < 0.05 vs. CS; *n* = 5–6).

To examine the anti-inflammatory activities of PPY, we evaluated level of proinflammatory cytokines (TNF-α, IL-6) in Bronchoalveolar Lavage (BAL) fluid. The levels IL-6 and TNF-α were significantly raised by CS exposure. CS exposure followed our previous chronic obstructive pulmonary disease (COPD)-experiment protocol (Figure 2). However, the levels of TNF-α and IL-6 were reduced significantly in the roflumilast and PPY (1, 2, and 10 mg/kg) treatment. PPY treatment decreased the levels of TNF-α and IL-6 in a dose dependent manner (Figure 3A,B). The concentrations of KC, neutrophil-chemoattractant chemokine, in BAL fluid were also measured with an ELISA. The expression of KC was increased in the CS group compared to the normal control (NC) group. Meanwhile, production of KC was declined in the roflumilast group and PPY (1, 2, and 10 mg/kg) groups compared to the CS group (Figure 3C).

### 2.2. PPY Inhibited the Recruitment of Inflammatory Cells into BAL Fluid

**Figure 4:**
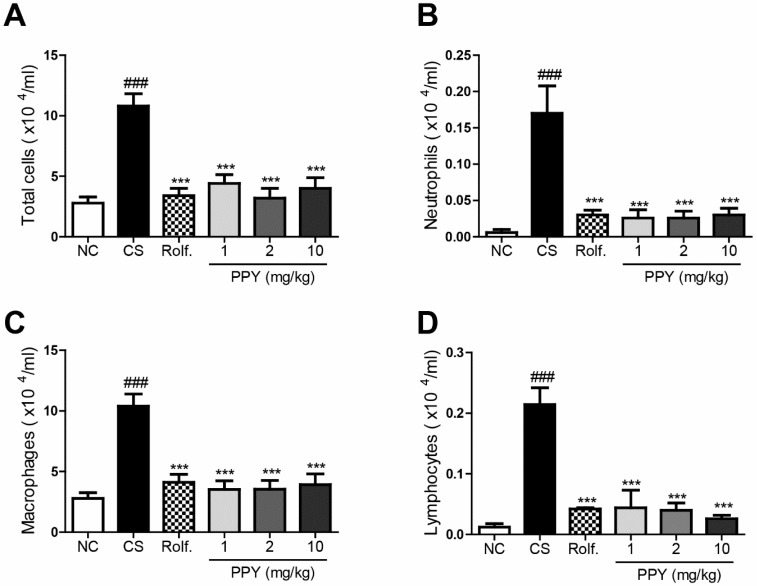
The effect of PPY on inflammatory cells in BAL fluid profiles. The numbers of cells in BAL fluid were counted using a hemocytometer, and differential cell counts were performed on slides prepared by cytocentrifugation at 250 rpm for 3 min followed by Diff-Quick staining. (**A**) Total cell number; (**B**) Macrophage count; (**C**) Neutrophil count; (**D**) Lymphocyte count. Data are shown as mean ± S.E.M. Statistical analyses were conducted by one-way ANOVA followed by Newman–Keuls Multiple Comparison test (### *p* < 0.001 vs. NC, *** *p* < 0.001 vs. CS; *n* = 5–6).

We next evaluated immune cell infiltrations in peribronchial airway. CS exposure significantly increased the numbers of lymphocytes, macrophages, neutrophils, and total cells in BAL fluid. However, oral administration of PPY (1, 2 and 10 mg/kg) or roflumilast markedly diminished the number of infiltrated immune cells including lymphocytes, macrophages, and neutrophils into lung tissue. The effect of PPY was comparable to that of roflumilast (Figure 4).

### 2.3. PPY Decreased the Morphological Change in Lung Tissue

**Figure 5:**
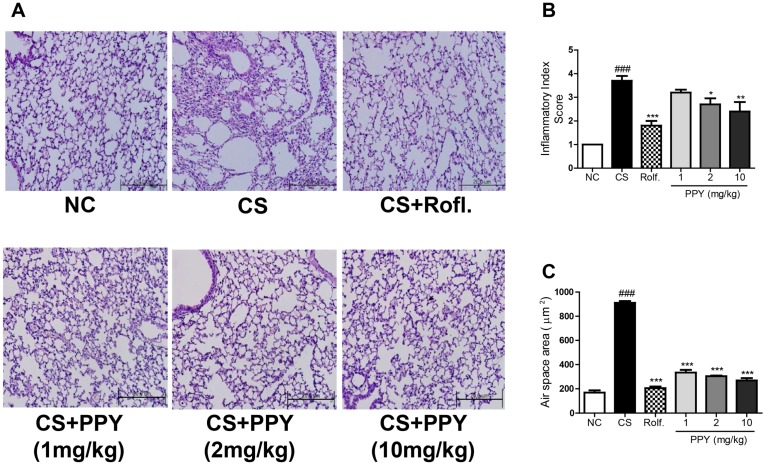
The effect of PPY on peribronchial inflammation and airspace enlargement in lung tissue (**A**) The right lower lobes of lung tissues were stained with H&E. to assess peribronchail inflammation (magnification 200×) as described in Materials and Methods; (**B**) Corresponding histopathological scores for lung inflammation. Peribronchial inflammation was evaluated on a subjective score 0 to 5 on blinded, randomized sections by three independent pathologists. All sections were scored from 0 to 5 according to the following criteria: 0 = normal; 1 = very mild; 2 = mild; 3 = moderate; 4 = marked; 5 = severe inflammation; (**C**) A morphometrical analysis for mean alveolar airspace was assessed using Image Pro-Plus 5.1 software as described in Materials and Methods. Data are shown as mean ± S.E.M. Statistical analyses were conducted by one-way ANOVA followed by Newman–Keuls Multiple Comparison test (### *p* < 0.001 vs. NC, *** *p* < 0.001, ** *p* < 0.01, * *p* < 0.05 vs. CS; *n* = 5–6).

To confirm the anti-inflammatory effects of PPY in histological feature, H&E staining was performed on the lung tissues (Figure 5A). Peribronchial region of the H&E stained tissued were ramdomly evaluated by patholosits. The histologic data showed that CS exposure caused peribronchial inflammation, whereas PPY or roflumilast treatment blocked it. The inflammatory index was significantly reduced in both the PPY group and the roflumilast group when compared to the CS group (Figure 5B). We also assessed the mean alveolar airspace to quantify the changes of airspace enlargement in the peribronchial regions. In the CS exposed mice, some alveoli were destroyed, resulting in enlarged air spaces indicating emphysematous changes. PPY- or roflumilast-treatment ameliorated the alveolar damages based on this quantitative assessment (Figure 5C).

### 2.4. Effect of PPY on Goblet Cell Hyperplasia

**Figure 6:**
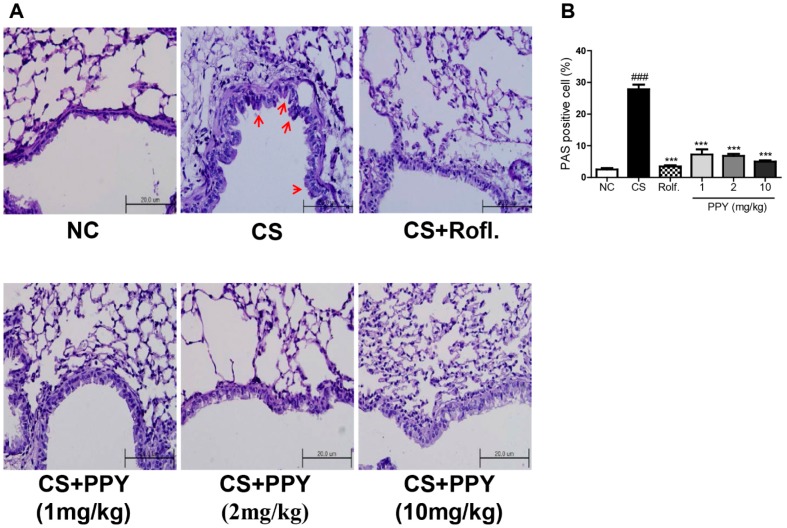
The effect of PPY on the histopathologic changes in lung. The right lower lobes of lung tissue were dissected and stained with periodic acid-Schiff (PAS). (**A**) The arrow indicates the PAS-positive cells (magnification 400×); (**B**) PAS-positive mucosal goblet cells around the bronchial airway were counted and are depicted as the percentage of goblet cells, as described in Materials and Methods. Data are shown as mean ± SEM. Statistical analyses were conducted by one-way ANOVA followed by Newman–Keuls Multiple Comparison test (### *p* < 0.001 vs. NC, *** *p* < 0.001 vs. CS; *n* = 5–6).

Periodic acid-Schiff (PAS) staining is used to evaluate goblet cell hyperplasia. We stained lung tissues with PAS to examine the anti-mucosal effect of PPY (Figure 6A). PAS stained cells were located around the peribronchial regions in the CS group, as expected. While, PPY treatment markedly decreased the percent of PAS-positive goblet cells around the bronchial airway (Figure 6B).

## 3. Discussion

Pathophysiological characters of COPD include inflammation, goblet cell hyperplasia, remodeling of the airways, and alveolar destruction [12]. Cigarette smoking is the most common risk factor in the development of COPD [13]. CS contains a large number of oxidants, and several adverse effects of CS may appear as the result of oxidative damage to critical biological substances and nuclear factor-κB (NF-κB) [14]. In addition, CS attacks lung tissue and activates lung-resident cells, including dendritic cells (DCs), alveolar macrophages, and epithelial cells, which release several inflammatory mediators that are capable of assembling inflammatory cells into lungs. Subsequently, this leads to a release of proinflammatory cytokines (TNF-α, IL-6) and chemotactic factors, antibacterial proteins, proteases, growth factors, and anti-inflammatory cytokines [15,16,17,18]. One of the main roles of alveolar macrophages is the secretion of chemotactic factors and this role is significantly increased by CS exposure. The enhanced release of chemotactic factors results in increased lung neutrophil infiltration, which is thought to be a key event in the development of COPD [19]. Furthermore, CXC chemokines such as IL-8, GRO-α, and KC play important roles in lung inflammation though they also have a chemotactic activity for alveolar macrophage [20,21].

Many previous studies have demonstrated that neutrophils, one of the assembled inflammatory cells induced by CS, increase in the sputum and lung tissue of patients with COPD and cause the recruitment of several types of inflammatory cells in the lung [18]. These immune cells secret proteases including matrix metalloproteinase-9, which leads to emphysema [22]. The morphological changes were also accompanied by an increase in lung lymphocytes, macrophages, macrophage-derived metalloproteases, neutrophils, neutrophil elastase, oxidants, IL-6, and TNF-α, all of which have been demonstrated to play significant roles in the pathogenesis of emphysema [23]. TNF-α is known as the activator of other proinflammatory cytokines including IL-6 and IL-8 [24]. TNF-α also induced NF-κB activation [25]. Also, IL-6 is a target in COPD as it triggers endothelial cell malfunction, leading to enlarged alveolar air spaces [26].

Though many medications are used for the treatment of COPD, side effects appear in several classes of drugs including steroids. For example, roflumilast is a widely used medication for COPD treatment. Nonetheless, roflumilast has dose-limiting adverse effects including weight loss, dizziness, insomnia, diarrhea, vomiting, headache, and nausea [27,28,29]. Thus, it is prudent that safer and more efficacious therapeutics for COPD are developed [30].

Natural herbs are successfully employed as anti-inflammatory agents with low adverse effects [30,31,32]. Therefore, they are considered as harmless alternatives to conventional medicines. Numerous research laboratories are currently using phytomedicines to control inflammatory responses. In the last ten years, more than 52 natural compounds, including chrysin were validated [33,34]. PPY is a new compound isolated from VF. Our previous study demonstrated that PPY blocked NF-κB activation in lung epithelial cells, resulting in a suppressed asthmatic response in ovalbumin-induced asthma. In addition, the pharmacological effect of PPY has been shown to be mediated by blocking activation of the NF-κB and the ERK1/2-signaling pathways and inhibiting the accumulation of eosinophil into the airway, and decreasing the levels of IL-4, IL-5, and IL-13 in BAL fluid. Based on the study, PPY has shown the potential as a novel therapeutic agent for allergic asthma [11]. The NF-κB pathway is an important drug target with regard to inflammation and metabolic dysfunction [35,36].

In this research, the effect of PPY on lung inflammation was examined in a CS-exposed mouse model. This model is preferred for research aiming to evaluate the degree of suppression of lung inflammation since it is caused by the equal factor and produces similar pathophysiological alterations including emphysema and airway remodeling with human COPD [37]. As a result, CS exposure raised the functional and structural changes, typical phenotypes of COPD in our model. The changes include increased levels of KC, IL-6, and TNF-α; goblet cell hyperplasia; airway remodeling; air space enlargement; and increased numbers of immune cells in the alveoli and airways. PPY treatment, on the other hand, inhibited these changes induced by CS exposure. PPY significantly decreased production of KC, IL-6, and TNF-α and suppressed immune cell infiltrations in the lung tissue. Reactive oxygen species (ROS) and toll-like receptors (TLRs) signals can also mediate airway inflammation. Zuo et al. have shown that increased level of pulmonary TNF-α can induce excessive ROS formation and targeting TLR cascade can be option for amelioration of airway inflammation [38,39]. Although effects of PPY on ROS and TLRs signaling are not unfold in this study, it is needed a detailed molecular mechanism of PPY in airway inflammation in further study. Besides, the role of IL-17 in pulmonary inflammation should not be underestimated. IL-17 signaling contributes for pulmonary immunopathology [40,41]. We tried to measure level of IL-17a in BAL fluid, however failed detection. The effect of PPY on IL-17 production in lung inflammation also should be evaluated in the future.

Histopathologic analysis confirmed that PPY treatment inhibits accumulation of inflammatory cells (especially neutrophils) and goblet cell hyperplasia throughout the peribronchial airway. Moreover, it is demonstrated that PPY treatment can protect from the structural changes induced by CS exposure through airspace quantitative assessment. In the study, however, we did not investigate the detailed mechanisms involved for these therapeutic effects. Experiments for validating the curative effects of PPY and conventional drug (ex. roflumilast) co-treatment are necessary to confirm the therapeutic benefits of PPY on COPD.

In conclusion, our data provide evidence that PPY has remarkable therapeutic benefits for CS-induced lung inflammation. Thus, we suggest PPY as a promising candidate for the treatment of lung inflammation. Further studies are required to elucidate detailed mechanisms and to develop the application to use PPY as a drug.

## 4. Materials and Methods

### 4.1. Reagent

PPY was isolated in the Institute for Korea Traditional Medical Industry (Gyeongsan, Korea). There, 3 kg of VF was extracted with MeOH (10 L × 5) by percolation for 24 h. To get a MeOH educt (100 g), the MeOH was evaporated in vacuo, then suspended in 1 L of H_2_O, and separated with *n*-BuOH (1 L × 3), EtOAc (1 L × 5), and *n*-hexane (1 L × 5). The EtOAc partition (17 g) was divided by silica gel using gradient mixtures of MeOH in CHCl_3_ (0→50%, and washed with MeOH 100%, 5 L each), affording PPY (0.0078% *w*/*w*, 235 mg). Roflumilast was dissolved in dimethylsulfoxide and then diluted with PBS.

### 4.2. Animal

The Kyung Hee University Animal Care and Use Committee approved the study protocol. The approval number is KHUASP (SE)-12-015 (10/10/2015). All experiments were conducted on 6 to 7 weeks old Balb/c female mice bought from Charles River Korea (Seungnam, Republic of Korea). They were kept under pathogen-free conditions with an air-conditioning, food provided ad libitum, and 12 h light/dark cycle.

### 4.3. Exposure to CS and Animal Treatment

CS exposure followed our previous COPD-experiment protocol (Figure 2) [42]. In brief, mice were divided randomly into six groups (*n* = 6~7/group). NC group: mice were exposed to only fresh air in the same duration of exposure to CS; CS group: mice were exposed to cigarette smoke from 3R4F reference cigarette (University of Kentucky, Lexington, KY, USA) for two weeks with six cigarettes per day, on day 0, 1, 4, 5, 6, 7, 8, 11, 12, 13; roflumilast group: roflumilast (5 mg/kg) was injected orally 2 h before CS exposure; PPY (1, 2 and 10 mg/kg) group: PPY (1, 2 and 10 mg/kg) was injected orally 2 h before CS exposure. All animals were sacrificed 24 h later after the last CS exposure. The experiments were performed two times independently.

### 4.4. Analysis of Inflammatory Cell Profiles in BAL Fluid

One ml of PBS was slowly infused into the lungs and withdrawn three consecutive times (final volume 2–2.5 mL). BAL fluid was then centrifuged and the supernatants were stored at −80 °C ultralow freezer until further use. The total viable cells were counted using a hemacytometer. Approximately Five hundred cells were counted. Differential cell counts with trypan blue exclusion method were performed on slides prepared by cytospin preparations (Sandon, Waltham, MA, USA) and Diff-Quick staining.

### 4.5. ELISA Analysis of IL-6, TNF-α, and CXCL1/KC

Total protein content in BAL fluid was measured using the BCA kit (Pierce Biotechnology Inc., Rockford, IL, USA) according to the manufacturer’s instructions. The levels of TNF-α, IL-6, and CXCL1/KC in the BAL fluid were measured with a commercial enzyme immunoassay kit (Becton Dickinson Bioscience, San Jose, CA, USA for TNF-α and IL-6; R&D systems, Minneapolis, MN, USA for Mouse CXCL1/KC) following the manufacturers’ protocols.

### 4.6. Immunohistochemistry of Lungs

Lungs were fixed by 4% paraformaldehyde infusion. Sliced lungs in 4 μm were stained with H&E sequentially for histological examination. Randomly selected five sections were examined using Image Pro-Plus 5.1software (Media Cybernetics, Inc., Silver Spring, MD, USA). To observe hyperplasia of goblet cells in the bronchial airway, some sliced tissues were stained with periodic acid Schiff (PAS).

### 4.7. Statistical Analysis

All values are presented as mean ± SEM. The statistical significance between study groups were assessed by one-way ANOVA followed by Newman–Keuls multiple comparison test using Prism 5.01 software (GraphPad Software Inc., San Diego, CA, USA). *p* < 0.05 was considered to be statistically significant.
